# The Multifaceted Functions of TRPV4 and Calcium Oscillations in Tissue Repair

**DOI:** 10.3390/ijms25021179

**Published:** 2024-01-18

**Authors:** Dongsheng Jiang, Ruiji Guo, Ruoxuan Dai, Samuel Knoedler, Jin Tao, Hans-Günther Machens, Yuval Rinkevich

**Affiliations:** 1 Institute of Regenerative Biology and Medicine, Helmholtz Center Munich, 81377 Munich, Germany; ruiji.guo@helmholtz-munich.de (R.G.); ruoxuan.dai@helmholtz-munich.de (R.D.); samknoe@gmail.com (S.K.); 2 Department of Plastic and Hand Surgery, Klinikum Rechts der Isar, School of Medicine, Technical University of Munich, 81675 Munich, Germany; hans-guenther.machens@mri.tum.de; 3 Division of Plastic Surgery, Brigham and Women’s Hospital, Harvard Medical School, Boston, MA 02152, USA; 4 Department of Physiology and Neurobiology and Centre for Ion Channelopathy, Medical College of Soochow University, Suzhou 215123, China; taoj@suda.edu.cn; 5 Jiangsu Key Laboratory of Neuropsychiatric Diseases, Soochow University, Suzhou 215123, China

**Keywords:** TRPV4, calcium oscillations, tissue repair, fibrosis

## Abstract

The transient receptor potential vanilloid 4 (TRPV4) specifically functions as a mechanosensitive ion channel and is responsible for conveying changes in physical stimuli such as mechanical stress, osmotic pressure, and temperature. TRPV4 enables the entry of cation ions, particularly calcium ions, into the cell. Activation of TRPV4 channels initiates calcium oscillations, which trigger intracellular signaling pathways involved in a plethora of cellular processes, including tissue repair. Widely expressed throughout the body, TRPV4 can be activated by a wide array of physicochemical stimuli, thus contributing to sensory and physiological functions in multiple organs. This review focuses on how TRPV4 senses environmental cues and thereby initiates and maintains calcium oscillations, critical for responses to organ injury, tissue repair, and fibrosis. We provide a summary of TRPV4-induced calcium oscillations in distinct organ systems, along with the upstream and downstream signaling pathways involved. In addition, we delineate current animal and disease models supporting TRPV4 research and shed light on potential therapeutic targets for modulating TRPV4-induced calcium oscillation to promote tissue repair while reducing tissue fibrosis.

## 1. Introduction

Transient receptor potential vanilloid type 4 (TRPV4) ion channels are calcium ion (Ca^2+^) permeable nonselective cation channels that belong to the transient receptor potential vanilloid (TRPV) subfamily of cation channels [1,2].

The original TRP channel was found in *Drosophila* photoreceptors. It contributes to sustained retinal depolarization when exposed to light. Deletion of the trp gene results in a transient calcium response, which lends its gene name. Unlike in *Drosophila*, mammalian TRPs serve a wider range of physiological functions [3,4]. In general, TRPs enable cells to detect subtle alterations in the local environment (e.g., fluid shear stress and mechanical forces), perceiving and responding to a variety of external cues through varied sensory perceptions, including vision, smell, taste, hearing, thermosensation, and most importantly, mechanosensation [5]. They are a class of ion channels found in diverse tissues and cell types, widely distributed in human organs, such as the liver, lungs, skin, kidneys, nerves, and intestines. They are permeable to a broad range of cations such as Ca^2+^, Mg^2+^, K^+^, Na^+^, and others [6]. TRP family members are categorized into two main groups, based on their sequence topology and phylogeny. Group 1 consists of TRPC (canonical), TRPV (vanilloid), TRPM (melastatin), TRPN (nompC), and TRPA (ankyrin), whereas group 2 includes TRPP (polycystin) and TRPML (mucolipin), totaling seven subfamilies [7]. Group 1 TRPs share substantial homology across their transmembrane domain sequence. Most of the group 1 TRP proteins are characterized by a C-terminal TRP domain, composed of 23 to 25 amino acids and located near the sixth transmembrane domain. In contrast, group 2 TRPs feature low sequence similarity with group 1, and an extracellular loop that is interposed between the first two transmembrane domains [2,5].

TRPV channels (TRPV1–TRPV6) exhibit diverse gating mechanisms. These versatile proteins serve as thermal, osmotic, and chemical sensors in vertebrates, hygrosensors, and mechanical sensors in insects, and function as both mechanical and chemical sensors in nematodes [8]. Notably, TRPV1 and TRPV2 are predominantly expressed in neurons but can also be detected in non-neuronal tissues [9,10,11]. Conversely, TRPV3 is expressed in the skin, hair follicles, tongue, nose, sensory ganglia, brain, and colon, while TRPV4 exhibits a broader expression pattern, including the skin [8,12].

TRPV4 was initially named on the basis of its distinct features, such as vanilloid receptor-related osmotically activated channel (VR-OAC) [13,14], osmosensitive transient receptor potential channel 4 (OTRPC4) [15], vanilloid receptor-like protein (VRL-2) [16], and TRP-12 [17]. In mammals, it was first identified as an osmotic detector, contributing to the maintenance of the systemic osmotic equilibrium [14,15,16]. Reduction of extracellular osmolarity conveyed by hypotonic solution, even as little as 10%, could trigger a reversible Ca^2+^ increase via TRPV4 in TRPV4-transfected HEK293 cells. Vice versa, when exposed to hypertonic environments, a decrease in Ca^2+^ was observed [15]. Notably, activation via heat stimuli has also been reported [13,18]. Temperature fluctuations between 36 °C and 42 °C were found to induce current fluctuations, while sustained or repeated stimulation over 42 °C resulted in TRPV4 desensitization and a decline in the current amplitude [18,19]. In the last decade, research on TRPV4 has mainly focused on its mechanosensory function, which is involved in various physiological and pathological processes, such as vasoconstriction [20], gastrointestinal motility [21], hair growth cycle [22,23], fibrosis [24,25,26], scleroderma [27], hypertension [28,29], gastric cancer [30,31], rosacea [32,33], brain injury [34], and pancreatitis [35,36].

## 2. Protein and Channel Structure of TRPV4

**Figure 1:**
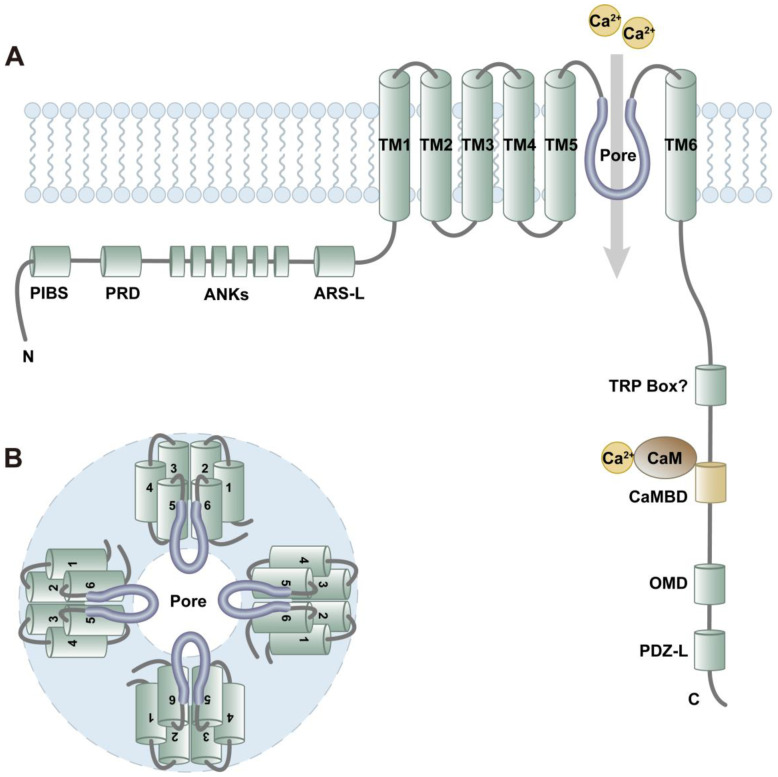
Protein and channel structure of TRPV4. (**A**) Protein structure of TRPV4. TRPV4 contains the N-terminal part, six transmembrane domains, and the C-terminal part. Ca^2+^-calmodulin complex binds to the calmodulin-binding domain. The fifth and sixth transmembrane domains (TM5, TM6) form the ion-conduction pore. (**B**) Structure of TRPV4 channel consisting of four monomers. PIBS: phosphoinositide-binding site. PRD, proline-rich domain; ANKs, ankyrin repeats; ARS-L, arachidonate-like recognition sequence; TM1–TM6, transmembrane domains; CaMBD, calmodulin-binding domain; OMD, oligomerization domain; PDZ-L, PDZ-like domain.

TRPV4 is a tetrameric non-selective cation channel that is not voltage-gated. Murine TRPV4 is comprised of 871 amino acids (aa). Both N-and C-terminal tails are located intracellularly, with six transmembrane segments spanning the cell membrane and forming the ion channel pore [37]. The structure of TRPV4 (from N to C terminal regions) includes a phosphoinositide-binding site (PIBS, aa121–aa125) [38], a proline-rich domain (PRD, aa132–aa144) [39], six ankyrin repeats (ANK1–6) [40], an arachidonate-like recognition sequence (ARS-L, aa402–aa408) [41], six transmembrane domains (TM1–TM6), a questioned TRP box [42], a calmodulin-binding domain (CaMBD, aa812–aa831) [43], an oligomerization domain (OMD, aa828–aa844) [44], and a PDZ-like domain (PDZ-L, last four aa) [43], which are situated in tandem [25] (Figure 1A). Typically, Ca^2+^ does not act directly on the channel but rather through attachment to the Ca^2+^-binding protein calmodulin (CaM). The Ca^2+^-CaM complex then binds to the C-terminal calmodulin-binding site. Accordingly, the deletion of the calmodulin-binding domain results in lack of Ca^2+^-dependent potentiation and slower ion channel activation [45]. Previous studies have revealed that inositol 1,4,5-trisphosphate (IP_3_) sensitizes TRPV4 to mechanical and osmotic stimulation. This sensitization process requires binding of the IP_3_ receptor (IP_3_R) to the TRPV4 calmodulin-binding site [43].

Four identical TRPV4 monomers form a functional channel. The transmembrane segments TM1–TM4 form a structural unit surrounding the central ion-conduction pore that is comprised of TM5 and TM6 [37] (Figure 1B). The selectivity filters in TRPV channels generally function through two barriers, namely the lower and upper gates. However, in comparison to other TRPV channels, the diameter at the narrowest point of the upper gate within the TRPV4 pore is larger than that of hydrated cations such as K^+^, Na^+^, and Ca^2+^. This implies non-selective entry/exit of cation ions in TRPV4 [37]. Due to the two negatively charged aspartate residues within the conserved lower gate, it is easier for divalent ions than monovalent ions to pass through TRPV4. As a result, TRPV4 is mainly permeable to Ca^2+^ rather than other ions, with the following permeability sequence: Ca^2+^ >> Mg^2+^ > K^+^ > Na^+^, whereby the permeability for Ca^2+^ is about five times higher than that of Mg^2+^, and about ten times higher than for Na^+^ [46,47].

## 3. TRPV4 Signaling in Mechanotransduction

**Figure 2:**
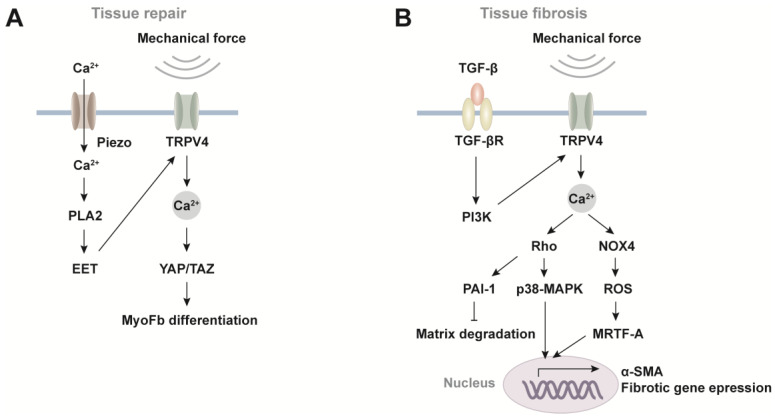
TRPV4 mediated signaling pathways. (**A**) TRPV4 signaling in tissue repair. Ca^2+^ influx through Piezo channel activates PLA2 with elevated production of EET. EET sensitizes TRPV4 activation in response to mechanical cues. TRPV4-evoked Ca^2+^ influx and subsequent Ca^2+^ oscillation activate downstream YAP/TAZ to promote myofibroblast (MyoFb) differentiation. (**B**) TRPV4 signaling in tissue fibrosis. TGF-βR activation induces PI3K, leading to Ca^2+^ influx via TRPV4. Ca^2+^ signal further activates Rho/Rho kinases, inhibiting matrix degradation via PAI-1, and upregulating fibrotic gene expression via p38-MAPK. TRPV4-induced Ca^2+^ signal also interacts with NOX4 to promote ROS production and nuclear translocation of MRTF-A, the coactivator of α-SMA expression. PLA2, phospholipase A2; EET, epoxieicosatrienoic acids; YAP/TAZ, yes-associated protein/transcriptional coactivator; MyoFb, Myofibroblast; PI3K, phosphatidylinositol 3-kinase; PAI-1, plasminogen activator inhibitor 1; MAPK, mitogen-activated protein kinases; NOX4, NADPH Oxidase 4; ROS, reactive oxygen species; MRTF-A, myocardin-related transcription factor A.

As a critical mechanotransduction molecule, TRPV4 plays a pivotal role in injury repair by orchestrating a wide array of cellular responses to mechanical stimuli (Figure 2A). For instance, in chondrocytes, TRPV4 is responsible for mechanotransduction in response to mechanical loading, together with Piezo channels. Specifically, TRPV4 and Piezo channels synergistically mediate calcium oscillation induced by different intensities of stretch stimulation. TRPV4-mediated Ca^2+^ signaling functions in response to low strain levels, whereas Piezo2-mediated Ca^2+^ signaling is activated in high strain settings, thus specifying the distinct cellular instructions during homeostasis and traumatic states [48,49]. Moreover, TRPV4 and Piezo1 can form a mechanosensory Ca^2+^ circuit. In pancreatic acinar cells, Piezo1 directly senses fluid shear stress and triggers Ca^2+^ influx, leading to an increase in phospholipase A2 (PLA2) activity and subsequent production of the arachidonic acid metabolites, particularly epoxieicosatrienoic acids (EET). EET sensitizes TRPV4 to initiate calcium oscillation, thereby amplifying the mechanical stimuli [36,43]. In response to changes in environmental stiffness, yes-associated protein/transcriptional coactivator with PDZ-binding motif (YAP/TAZ) acts as a downstream molecule for TRPV4 activity. This activation leads to myofibroblast differentiation, which plays a significant role in wound healing and injury repair [50,51].

TRPV4 is also involved in tissue fibrosis processes (Figure 2B). The most extensively studied fibrotic pathways mediated by TRPV4 are found in pulmonary and cardiac fibroblasts. Following acute lung injury repair, there is an abundance of TGF-β1 in the environment. TRPV4 activation is sustained in lung fibroblasts through TGF-β1 and its downstream phosphatidylinositol 3-kinase (PI3K) pathway. Consequently, Ca^2+^ signaling activates Rho family GTPases, which further activates p38-MAPK to upregulate fibrotic gene expression, and induces plasminogen activator inhibitor 1 (PAI-1) to reduce matrix degradation [52,53]. The imbalance of synthesis and degradation leads to the accumulation of extracellular matrix (ECM) and tissue stiffening, which provides a positive feedback loop for TRPV4 activity. In addition, through interaction with NADPH Oxidase 4 (NOX4), TRPV4 in lung fibroblasts potentiates the production of reactive oxygen species (ROS) to promote nuclear translocation of myocardin-related transcription factor A (MRTF-A), a transcription coactivator of α-SMA [26,52]. Therefore, TRPV4 stands at the crossroads of fibrotic signaling pathways that integrate the mechanical, TGF-β1, and ROS signals to promote myofibroblast differentiation of lung fibroblasts, by remodeling both intracellular actomyosin and ECM. Similar responses and signaling pathways have been documented in human ventricular cardiac fibroblasts and *Trpv4*-deficient mice. TRPV4-mediated Ca^2+^ influx regulates cardiac fibrotic gene promoter activity and myofibroblast differentiation [54] through the Rho/Rho kinase pathway activation and the mechanosensitive transcription factor MRTF-A [55], and the downstream MAPK/ERK pathway [56]. These findings indicate that TRPV4-mediated Ca^2+^ signaling is crucial for injury repair, but uncontrolled and persistent signaling results in fibrosis and scarring following the healing process.

## 4. TRPV4 Triggers Ca^2+^ Oscillations

Since cytosolic Ca^2+^ participates in numerous cellular activities, such as migration, contraction, exocytosis, cell growth and cell death [57], Ca^2+^ is strictly maintained in specific regions, normally at concentrations of less than a few hundred nanomolar. It can reach micromolar concentrations when Ca^2+^ is mobilized for cellular activities [58]. In essence, there are two reservoirs for Ca^2+^: (i) external Ca^2+^ within the extracellular microenvironment and (ii) sequestered Ca^2+^ within organelles, for instance, endoplasmic reticulum (ER) [58].

**Figure 3:**
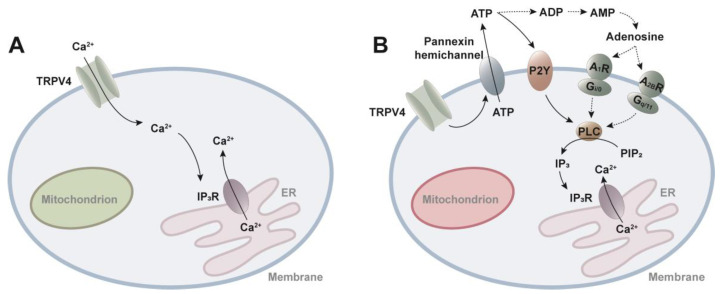
TRPV4-evoked calcium oscillations. (**A**) Ca^2+^ oscillation induced by TRPV4 under normal circumstances. TRPV4-mediated Ca^2+^ influx activates IP_3_R at ER membrane, which controls Ca^2+^ release and uptake from intracellular Ca^2+^ storage. (**B**) When the mitochondrial membrane potential is impaired due to increased ROS level, TRPV4 activation provokes ATP release through pannexin hemichannels. Released ATP binds P2Y receptors or adenosine resulted from ATP degradation bounds to G_i/0_-coupled A_1_R or G_q/11_-coupled A_2B_R, activates PLC, which converts PIP_2_ to IP_3_. IP3 binds to IP_3_R channels on ER initiating calcium oscillation. IP_3_, 1,4,5-trisphosphate; IP_3_R, IP_3_ receptor; P2Y: purinergic receptor; PLC, phospholipase C; PIP_2_, Phosphatidylinositol 4,5-bisphosphate; A_1_R: adenosine A_1_ receptor. A_2B_R: adenosine A_2B_ receptor.

Ca^2+^ influx from outside the cell via TRPV4 evokes Ca^2+^ release from internal storages, termed “Ca^2+^-induced Ca^2+^ release”. This process is mediated through IP_3_R calcium ion channels in the ER of astrocytic endfeet, causing repetitive Ca^2+^ oscillations [59] (Figure 3A). When extracellular Ca^2+^ is removed, intracellular Ca^2+^ responses in chondrocytes can be completely suppressed, highlighting the necessity of extracellular Ca^2+^ for the onset of Ca^2+^ signaling [60]. Of note, the same phenomenon is also found in pulmonary fibroblasts [61]. Under abnormal circumstances, the equilibrium of mitochondrial membrane potential can be disrupted by elevated ATP production, thermogenesis, or mitochondrial dysfunction [62], including factors such as hyperoxia and pharmacologic uncoupling of mitochondrial oxidative phosphorylation [63,64]. These conditions lead to an increase in the levels of reactive oxygen species (ROS) surrounding the mitochondria [65]. Consequently, the release of Ca^2+^ is triggered through a more intricate pathway (Figure 3B). TRPV4 activation induces ATP release through pannexin hemichannels, whereupon ATP stimulates plasma membrane-bound purinergic receptors (P2Y), which activate phospholipase C (PLC) to produce IP_3_, eventually culminating in the activation of IP_3_R Ca^2+^ channels [64]. Moreover, ATP released through pannexin into the extracellular space undergoes degradation into ADP, AMP, and adenosine by ectonucleoside triphosphate diphosphohydrolase (ENTDPase) and ecto-5′-nucleotidase (ecto-5′-NT) [66,67]. Adenosine, a key player in this process, exerts its effects by binding to four distinct receptor subtypes—A_1_R, A_2A_R, A_2B_R, and A_3_R—belonging to the G-protein coupled receptor (GPCR) family [68]. When A_1_R is coupled to the G_i/0_ proteins and A_2B_R is coupled to the G_q/11_ proteins, the stimulation of PLC promotes the cleavage of phosphatidylinositol 4,5-bisphosphate (PIP_2_) into diacylglycerol (DAG) and IP_3_, which in turn increase intracellular Ca^2+^ levels [69]. In addition to the modulation of mitochondrial membrane potential, it has been documented that TRPV4 activation in hippocampal neurons leads to the depolarization of the resting membrane potential. Notably, heat stimulation induced depolarization exclusively in TRPV4-positive neurons, while TRPV4-negative or TRPV4-KO neurons did not exhibit such changes [70,71]. Furthermore, TRPV4 activation through hypotonic stimulation or the TRPV4 agonist 4α-PDD initiates NMDA-activated currents in hippocampal CA1 pyramidal neurons, with the inward current demonstrating a dose-dependent relationship to 4α-PDD [72]. Simultaneously, TRPV4 activation inhibits GABA-activated currents and increases glycine-activated currents [73,74].

It is worth noting that cytosolic Ca^2+^ oscillations correlate with TGF-β [75,76], platelet-derived growth factor (PDGF) [61] and ATP [58]. PDGF also requires PLC and IP_3_-gated channels in its downstream to induce Ca^2+^ waves [61]. The oscillatory frequency is associated with growth factor concentrations [76]. More importantly, calcium oscillation is necessary for proper cellular functions, since sustained high levels of Ca^2+^ induce cell apoptosis via PERK/CHOP/Bcl2 apoptotic pathway [77].

The downstream effects of calcium oscillation are believed to be mediated by second messengers. Yet, to date, only a few second messenger pathways have been identified, and they typically depend on the spatial and temporal context. To achieve its precision in modulation, Ca^2+^ oscillation—as a common form of Ca^2+^ signals—is encoded by the amplitude and frequency of Ca^2+^ spikes [57]. Once the amplitude is modulated, for example if the affinity of the decoder to the Ca^2+^ binding site is high, it is sensitive to the subtle Ca^2+^ elevations in the cytoplasm. Conversely, if the decoder’s affinity to the Ca^2+^ binding site is low, a more significant increase in pathway activation is required. In frequency modulation, Ca^2+^/calmodulin-dependent protein kinase II (CaMKII), a downstream Ca^2+^ sensor, fails to respond to low-frequency Ca^2+^ oscillations. In contrast, when the frequency of calcium oscillations exceeds its threshold, the kinase activity increases in a frequency-dependent manner [78].

The finely synchronized interplay of amplitude and frequency of Ca^2+^ signals in calcium oscillation is highly relevant in signal transduction and cellular function, with Ca^2+^ oscillation providing more information compared to a single pulse. For example, the amplitude and frequency of Ca^2+^ oscillations in chondrocytes are directly linked to the viscoelastic properties during cell swelling on substrates of different stiffness [79]. Lung fibroblasts exhibit recurring Ca^2+^ transients in response to PDGF, which spread through the cells as Ca^2+^ waves. There is a sigmoidal relationship between the frequency of Ca^2+^ wave and PDGF concentration [61]. Trabecular meshwork cells are specialized cells in the eye, responsible for regulating fluid outflow in the anterior portion of the eye. Long-term stimulation on TRPV4 is required to trigger Ca^2+^ oscillation in trabecular meshwork cells. Via the Ca^2+^ peak phase, plateau phase, and fluctuation phase, the mechanical signal can be transduced via trabecular meshwork cells to regulate intraocular pressure, thus upholding healthy fluid balance and pressure within the eye [80].

## 5. Function of TRPV4-Evoked Ca^2+^ Oscillations in Injury Repair and Fibrosis

**Figure 4:**
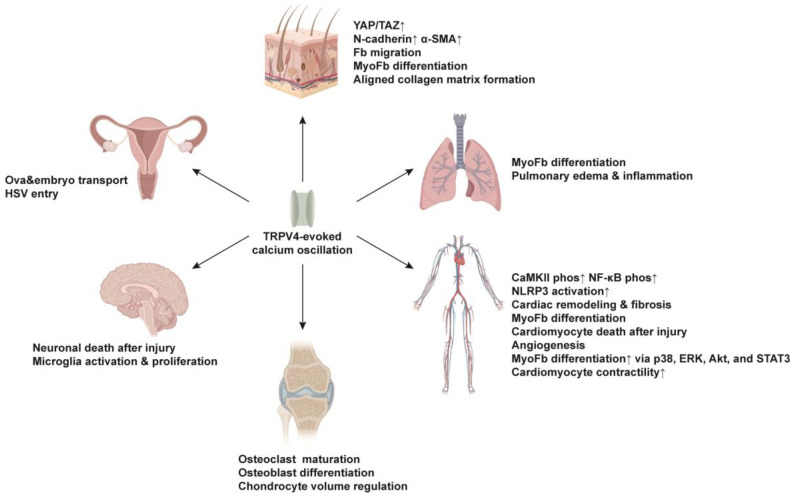
Functions of TRPV4-induced Ca^2+^ oscillations across different organs. TRPV4 is essential for physiological activities in the skin, lung, cardiovascular, skeletal, nervous and reproductive systems. Fb, fibroblast; MyoFb, myofibroblast; Phos, phosphorylation; HSV, Herpes simplex virus. Upward arrows indicate higher expression or activity.

TRPV4 mechanosensor is an integral factor in the progression of tissue repair and fibrotic disorders across different organs, including the skin, lungs, liver, kidneys, brain, blood vessels and heart [47]. In this section, we provide a comprehensive summary of the current understanding regarding the functions of TRPV4-induced Ca^2+^ oscillation in each specific organ (Figure 4). It is noteworthy that the mechanisms underlying cytosolic calcium oscillations in excitable and non-excitable cells significantly differ in the context of TRPV4 activation. Essential details, including whether the cells are excitable or non-excitable, are summarized in Table 1. This review primarily centers on examining the influence of TRPV4-induced Ca^2+^ oscillation in non-excitable cells during tissue repair and fibrosis.

### 5.1. Skin

In a bleomycin-induced skin fibrosis model, increasing matrix stiffness was found to activate TRPV4, with TRPV4 being essential for the nuclear translocation of YAP/TAZ in response to matrix stiffness. Genetic ablation of TRPV4 (*Trpv4*-knockout) or pharmacological inhibition of TRPV4 could ameliorate this fibrotic response. The authors attributed the effect of TRPV4 to its role in regulating the process of epithelial-mesenchymal transition [51]. In this study, it was also found that TRPV4-induced Ca^2+^ oscillations led to higher expression of N-cadherin and α-SMA on skin fibroblasts [51]. Our group has recently revealed that N-cadherin [109]—together with Connexin-43 [110] and p120-catenin [111]—are critical intercellular adhesion molecules that coordinate the collective migration of fibroblasts at a supracellular level needed for wound repair and subsequent scar formation. Therefore, in the skin fibrosis model, TRPV4′s effect on fibrosis development could also be attributed to its induction of fibroblasts’ collective migration and cell state transition toward myofibroblasts. However, the detailed signaling components and routes between TRPV4-induced Ca^2+^ oscillations and YAP/TAZ nuclear translocation, and the role of Ca^2+^ oscillations in synchronizing collective fibroblast migrations are yet to be determined. Of note, TRPV4-induced Ca^2+^ oscillations seem to be involved in adipogenic differentiation. Knocking down *TRPV4* using shRNA significantly reduced adipogenesis in human preadipocytes via reduced phosphorylation of Akt kinase [81].

In addition, TRPV4-mediated Ca^2+^ oscillations are also implicated in matrix deposition. Gilchrist et al. found that the TRPV4 agonist GSK1016790A induced Ca^2+^ oscillation in mesenchymal stem cells at low concentrations (1, 10 nM), and is required for collagen matrix assembly in an aligned manner [82]. Interestingly, at a higher concentration (100 nM), the agonist initially activated TRPV4 and yielded a strong Ca^2+^ influx, but failed to sustain a rhythmic Ca^2+^ oscillation and regulate the formation of aligned collagen matrix [82].

TRPV4 is expressed in the hair follicles during the growing anagen phase. More specifically, it can be detected mainly in the outer and inner root sheath layers of the hair follicle epithelium, particularly in keratinocytes. The activation of TRPV4 through its agonist GSK1016790A was found to cause a significant reduction in hair shaft elongation and induce morphological and structural changes in anagen hair follicles, pushing them toward the catagen phase [23]. This study indicates that TRPV4-induced Ca^2+^ influx promotes injury repair and fibrotic processes, yet it may also suppress hair follicle regeneration after wounding. Therefore, targeting TRPV4 could either facilitate skin wound healing by enhancing Ca^2+^ influx, or stimulate hair follicle regeneration and reduce scarring by blocking its effects.

### 5.2. Lung

In human pulmonary fibroblasts, activation of TRPV4 assists cell membrane depolarization. When the membrane potential is in the voltage range of voltage-dependent Ca^2+^ current, the activated Ca^2+^ influx contributes to Ca^2+^ oscillations [76]. TRPV4 has also been reported to mediate TGF-β evoked repetitive Ca^2+^ waves in human lung fibroblasts, leading to the upregulation of ECM genes encoding collagen and fibronectin, and increased phosphorylation of SMAD-2 protein [75]. Via the P2Y receptor, ATP is able to release internal Ca^2+^ through ryanodine-insensitive channels, thereby exerting its effect on Ca^2+^ waves [58].

TRPV4 activity has been found to be significantly higher in patients with idiopathic pulmonary fibrosis. Activation of TRPV4 in lung fibroblasts accelerates the differentiation of myofibroblasts and contributes to the progression of pulmonary fibrosis [26]. In this context, it is worth mentioning that TRPV4 expression on murine lung fibroblasts is necessary for TGF-β-induced myofibroblast differentiation. Furthermore, TRPV4 mechanotransduction increases on stiffer matrices or in fibrotic lung tissue, while *Trpv4*-deficient mice are protected from bleomycin-induced lung fibrosis [26,54]. These findings imply that TRPV4 antagonists may represent potential targets for pulmonary fibrosis disease.

While TRPV4 activation promotes skin wound repair, its activity in lung endothelial cells has been shown to be detrimental in the setting of lung ischemia-reperfusion (IR) injury. In fact, TRPV4 activation in lung endothelial cells increases vascular permeability, thus causing increased lung inflammation and edema after IR [83]. Genetic depletion of TRPV4 using *Trpv4^−/−^* mice or pharmacological inactivation of TRPV4 with the TRPV4-specific inhibitor GSK2193874a markedly reduced pulmonary edema and neutrophil infiltration after IR injury, when compared to untreated wild-type mice. To further investigate TRPV4′s role in endothelial cells, Ottolini et al. generated an inducible endothelial *Trpv4* knockout line by crossing *Trpv4* floxed mice (*Trpv4^fl/fl^*) with tamoxifen-inducible VE-Cadherin (*Cdh5*) Cre mice [84]. The endothelial-specific *Trpv4* knockout exhibited the same phenotype as observed in the whole-body *Trpv4* knockout, suggesting that TRPV4 activity in endothelial cells contributes to pulmonary dysfunction and edema following IR injury. In line with the IR injury model, inhibition of TRPV4 had a protective effect in a mouse model of resistive breathing (RB)-induced lung injury. During RB, increased mechanical stress is imposed on the lung, thus leading to lung injury. The administration of the TRPV4 antagonist HC-067047 significantly mitigated symptoms by restoring static compliance and ameliorating the lung inflammation by reducing the number of macrophages and neutrophils, and the inflammatory chemokines keratinocyte-chemoattractant (KC) and interleukin (IL)-6 in bronchoalveolar lavage [112].

### 5.3. Cardiovascular System

The role of TRPV4 in cardiac remodeling has been investigated in the context of myocardial infarction and subsequent fibrosis, with a focus on TRPV4 channels expressed in non-excitable cardiac fibroblasts. Experimental findings demonstrate that while hypotonicity induces significant Ca^2+^ influx in cardiac fibroblasts from wild-type mice, this response is markedly diminished in cardiac fibroblasts derived from *Trpv4*-knockout mice, thereby establishing TRPV4′s role as a mechanosensor in fibroblasts [55]. In vitro studies using rat or human cardiac fibroblasts have revealed that TRPV4 activation by specific agonists such as 4αPDD or GSK1016790A leads to Ca^2+^ influx, which subsequently promotes the differentiation of fibroblasts into myofibroblasts through the p38 and ERK MAPK pathways [56,85]. Consistently, the blockade of TRPV4 by different antagonists or *Trpv4*-siRNA inhibits TGFβ-induced myofibroblast transformation by inhibiting the p38, Akt, and STAT3 signaling pathways [86,87]. In addition, it has been noticed that TRPV4 protein expression increases in cardiomyocytes of the aged heart. TRPV4-evoked calcium oscillations are directly correlated to the contractility of cardiomyocytes and contribute to tissue damage in the aged heart following ischemia-reperfusion (I/R), a pathological condition associated with cardiomyocyte osmotic stress. TRPV4 antagonist HC-067047 is effective in preventing Ca^2+^ overload in cardiomyocytes after I/R and prevents hypoosmotic stress-induced cardiomyocyte death and I/R-induced cardiac damage in the elderly population [91,92]. The same observation is documented in the aged *Trpv4* knockout mice [93]. Furthermore, TRPV4-induced Ca^2+^ oscillation has been reported in human cardiac c-kit^+^ progenitor cells, regulating their migration but not proliferation [88]. In endothelial colony-forming cells, TRPV4-mediated Ca^2+^ oscillation results in the nuclear translocation of a Ca^2+^-sensitive transcription factor p65 NF-κB and induces angiogenesis [89].

In addition, TRPV4 channel expression is found to be significantly increased in failing human ventricles, and in murine ventricles using a mouse model of pressure overload-induced cardiac hypertrophy [90]. Compared to wild-type mice, *Trpv4* knockout mice showed a remarkable reduction in cardiac hypertrophy, cardiac dysfunction, fibrosis, and inflammation. Those findings imply that TRPV4 is involved in cardiac remodeling and fibrosis after injury. Moreover, the absence of TRPV4 prevented an elevation of CaMKII phosphorylation in excitable cardiomyocytes of a pressure-overloaded left ventricle. It has been proposed that the TRPV4-mediated increase in CaMKII phosphorylation triggers NF-κB phosphorylation and NLRP3 activation, both of which contribute to the pro-inflammatory remodeling observed in these pressure-overloaded hearts [90].

In the vascular system, TRPV4 expression is widely reported in vascular smooth muscle cells (VSMCs), and TRPV4 plays an important role in the regulation of blood pressure and the development of hypertension. In patients with hypertension and in mouse models of hypertension, VSMCs showed elevated TRPV4 activity, resulting in signal amplification for the simulation of α1 adrenergic receptor. Strikingly, VSMC-specific *Trpv4*-knockout mice were protected from hypertension [95]. TRPV4 expression in VSMCs also contributed to vascular remodeling after injury, whereby TRPV4 was activated by platelet-derived microvesicles and triggered Ca^2+^ influx from the ECM and subsequent Ca^2+^ oscillations, thus contributing to the modulation of the cell migration [94].

### 5.4. Skeletal System

A mounting body of evidence indicates that TRPV4 is required for the normal development and maintenance of bone and cartilage. Osteoclasts are considered the main mediators of bone resorption. Their activity in removing damaged tissue and modulating the bone microenvironment is crucial for subsequent phases of bone healing and the restoration of bone structure and function. The maturation and function of osteoclasts are regulated by intracellular Ca^2+^ oscillatory signals, which are stimulated by mechanical forces, such as fluid shear stress. TRPV4 was found to be highly expressed in mature osteoclasts and necessary for Ca^2+^ oscillation induced by fluid shear stress [96]. Ca^2+^ oscillation affected the bone homeostasis-related gene expression, including sclerostin (*Sost*), osterix (*Sp7*), and osteoprotegerin (*Tnfrsf11b*) [97], and osteoblastic differentiation genes, such as alkaline phosphatase (*Alpl*), osterix (*Sp7*), dentin matrix protein 1 (*Dmp1*), and osteocalcin (*Bglap*) [98]. Spontaneous Ca^2+^ oscillation was generated by intracellular Ca^2+^ release as well as Ca^2+^ reuptake, and sustained by Ca^2+^ influx. It was gradually shifted to Ca^2+^ influx through TRPV4 during osteoclast differentiation, so that Ca^2+^ oscillations and Ca^2+^ influx via TRPV4 were sequentially required [99]. Upon exposure to an electromagnetic field, TRPV4 expression was downregulated in osteoclasts, and Ca^2+^ oscillations were impaired as a result of Ca^2+^ influx decrease, culminating in reduced activity of the calcium/calmodulin-dependent protein kinase–cyclic AMP response element-binding protein (CaMK–CREB) pathway [113].

In chondrocytes, intracellular Ca^2+^ oscillations are one of the earliest responses elicited by physical stimuli, such as compressive loading and stretch [60,114]. TRPV4 serves as a major mechanical sensor on chondrocytes by regulating Ca^2+^ signaling, together with Piezo1 and Piezo2 channels [100]. Evoked Ca^2+^ oscillation regulates chondrocyte volume in response to the pericellular matrix [79]. In stiff substrates, cytosolic Ca^2+^ oscillations were enhanced in the cell recovering phase, whereas in soft substrates, the cytosolic Ca^2+^ oscillations were increased in the cell swelling process. Of note, heterozygous mutations in *TRPV4* led to altered chondrocyte Ca^2+^ oscillation and caused severe metatropic dysplasia [114].

Intracellular Ca^2+^ oscillation and a sustained Ca^2+^ response have been documented in annulus fibrosus cells, which form one of the structural components of intervertebral discs. This suggests that TRPV4 is a crucial regulator of the perception of mechanical stimulation in the intervertebral discs of the spine [101]. Furthermore, TRPV4 is also implicated in joint inflammation. Both TRPV4 and TRPV1 are activated in synovial cells by protein kinase A and protein kinase C, leading to increased cell sensitivity to noxious thermal changes and hypoosmotic stress [102,103]. In contrast, in a mouse model of osteoarthritis, knockout *Trpv4* in articular chondrocytes (*Col2a1^CreER^*;*Trpv4^fl/fl^*) induced more severe age-related osteoarthritis [115].

### 5.5. Nervous System

TRPV4 is expressed in microglia, a type of non-excitable cells within the central nervous system (CNS), and plays a role in microglia activation and proliferation, promoting functional and structural plasticity in excitatory spinal cord neurons through the release of lipocalin-2 [104]. Genetic ablation of *Trpv4* in microglia using *Cx3cr1^CreER^*;*Trpv4^fl/fl^* mice or a pharmacological blockade of TRPV4 with TRPV4 antagonist GSK2193874 markedly attenuated neuropathic pain-like behavior in a mouse model of spared nerve injury [104], highlighting TRPV4 as a potential target for chronic pain treatment.

The functional involvement of TRPV4 in excitable neurons in the spinal cord remains a subject of controversy. Previous studies have reported TRPV4 expression in spinal dorsal horn neurons, ventral horn neurons, and dorsal root ganglion in Sprague-Dawley rats and neonatal mice, indicating a direct influence on the induction of neuropathic pain [116,117,118]. In contrast, recent work by Hu and colleagues challenges this perspective, asserting that TRPV4 is not expressed in NeuN^+^ spinal cord neurons but rather in IBA1^+^/Tmem119^+^ microglia and CD31^+^ endothelial cells, as revealed by genetic lineage tracing in mice. This suggests that TRPV4 might participate in pain signaling through sustained neurogenic inflammation [104].

TRPV4 also plays a crucial role in neuronal damage during cerebral ischemia. In a rat model of hypoxia/ischemia-induced cerebral injury, the expression of TRPV4 is markedly enhanced in hippocampal astrocytes. The increased activity of TRPV4 channels triggers Ca^2+^ oscillations in these cells, promoting active cellular proliferation that protects neurons, initiates repair, and correlates with astrogliosis—scar formation in the brain [105]. TRPV4 expression undergoes upregulation in the ipsilateral hippocampus following middle cerebral artery occlusion, contributing to neuronal injury during cerebral ischemia. The neurotoxic effects associated with TRPV4 activation coincide with an elevation in the phosphorylation of the NR2B subunit of the N-methyl-D-aspartate receptor (NMDAR) and a concurrent downregulation of the Akt signaling pathway. Consequently, the TRPV4-specific antagonist HC-067047 has proven effective in reducing cerebral infarction. Conversely, intracerebroventricular injection of the TRPV4 agonist GSK1016790A0 has been shown to induce hippocampal neuronal death [119]. In *Trpv4*-knockout mice, impaired osmotic sensing and regulation in the CNS have been observed. In this context, TRPV4-induced Ca^2+^ oscillations, which trigger c-FOS expression in osmotically responsive cells of the CNS, become dysfunctional [120].

Moreover, rapid Ca^2+^ oscillations are recorded in the subpial endfeet of astrocytes, with oscillatory activities relying on the IP_3_R type 2 channel correlating with TRPV4 and dystrophin. Astrocytes leveraged the Ca^2+^ response to effectively respond to hypoosmotic changes in the cerebrospinal fluid [106]. However, this particular function is lost after subarachnoid hemorrhage [121].

### 5.6. Reproductive System

TRPV4, in conjunction with γ-aminobutyric acid receptors A and B (GABA A/B), has been identified as a contributor to progesterone (P4)-induced ova and embryo transport. In the mouse oviduct, Ca^2+^ oscillation and an increase in ciliary beat frequency are seen in response to P4 and GABA A/B activation. Importantly, TRPV4-mediated Ca^2+^ entry is essential for the initiation and maintenance of this oscillatory signal [107]. In addition, during infection in vaginal epithelial cells, Ca^2+^ fluctuations seem to play a critical role in facilitating the entry of Herpes simplex virus (HSV). TRPV4 expressed in human epithelial cells mediates the influx of Ca^2+^. Inhibition of TRPV4 by TRPV4 inhibitors, such as GSK2193874 and HC067047, not only reduces HSV-2 infection in human vaginal epithelial cells, but also attenuates the associated inflammatory responses, characterized by the expression of tumor necrosis factor (TNF)-α, IL-6, C-X-C motif chemokine ligand (CXCL)-9, and CXCL-10 [108]. These findings underscore the potential of TRPV4 as a target for therapeutic interventions related to reproductive health and HSV-2 infection.

## 6. Conclusions and Future Perspectives

The body of evidence summarized herein highlights the multifaceted role of TRPV4-induced Ca^2+^ influx and of Ca^2+^ oscillations across different tissues and under different pathological conditions. Altogether, TRPV4-mediated calcium oscillations have emerged as a crucial regulatory mechanism in injury repair and tissue fibrosis. Understanding the precise role of TRPV4 in these processes may accelerate the development of innovative therapeutic approaches in a wide array of disorders.

Notably, the physiological and pathological functions of TRPV4-induced Ca^2+^ oscillations in skin wound repair and scarring remain relatively unexplored. Given its ramifications in fibroblast maturation, differentiation, cellular migration, ECM remodeling, and inflammation, it is tempting to speculate that TRPV4-mediated Ca^2+^ oscillations play a pivotal role in wound healing and scarring. Accordingly, future research endeavors should prioritize unraveling the precise underlying mechanisms and exploring targeted strategies that engage TRPV4 to promote effective skin wound healing and mitigate scar formation. In this context, the development of readily transferable research tools such as fibroblast subset-specific (e.g., papillary, reticular, hypodermal, superficial fascia) or fibroblast state-specific (e.g., naïve, proinflammatory, proto-myofibroblast and myofibroblast) TRPV4 knockout animals will be invaluable to decipher TRPV4-evoked Ca^2+^ oscillations in fibroblast fate determination. These avenues can help bridge the gap between basic science and clinical translation and, ultimately, optimize patient care.

In conclusion, by elucidating the interplay between TRPV4 and other cellular components, such as ion channels, receptors, and intracellular signaling molecules, we can gain a comprehensive understanding of TRPV4′s functional significance in diverse fibroblast subsets and cell states. This knowledge will guide the design and engineering of selective TRPV4 modulators that precisely regulate Ca^2+^ oscillation, which holds tremendous potential for revolutionizing wound healing treatments. The ability to fine-tune TRPV4-mediated Ca^2+^ oscillation could lead to improved outcomes and reduced scar formation.

## Figures and Tables

**Table 1:** The roles of TRPV4-evoked Ca^2+^ oscillations in injury repair and fibrosis across organs.

Organ	Species	Tissue/Cell Type	Non-Excitable/Excitable	State of TRPV4	Physiological/Pathological Function	References
Skin	mouse	skin fibroblasts	non-excitable	activation	nuclear translocation of YAP/TAZ, N-cadherin and α-SMA↑, myofibroblasts differentiation	[51]
	human	preadipocytes	non-excitable	activation	phosphorylation of Akt kinase↓, adipogenesis↓	[81]
	human	mesenchymal stem cells	non-excitable	activation	aligned collagen matrix assembly	[82]
Lung	human	lung fibroblasts	non-excitable	activation	ECM genes↑, collagen and fibronectin↑, phosphorylation of SMAD-2 protein↑	[75,76]
	mouse	lung fibroblasts	non-excitable	activation	differentiation of myofibroblasts↑, stiffer matrices, pulmonary fibrosis↑	[26]
	mouse	lung endothelial cells	non-excitable	activation	vascular permeability↑, lung inflammation and edema↑	[83,84]
Cardiovascular system	mouse	cardiac fibroblasts	non-excitable	inhibition	fibrosis↓ after myocardial infarction	[55]
	rat, human	cardiac fibroblasts	non-excitable	activation	via p38 and ERK MAPK pathways, fibroblasts differentiation↑, myofibroblasts↑	[56,85]
	rat	cardiac fibroblasts	non-excitable	inhibition	p38, Akt, and STAT3 signaling↓	[86,87]
	human	cardiac c-kit+ progenitor cells	non-excitable	inhibition	migration↓	[88]
	human	endothelial colony-forming cells	non-excitable	activation	nuclear translocation of a Ca^2+^-sensitive transcription factor p65 NF-κB, angiogenesis↑	[89]
	mouse, rat, human	cardiomyocytes	excitable	activation	CaMKII phosphorylation↑, NF-κB phosphorylation↑, NLRP3 activation↑	[90]
	mouse	cardiomyocytes	excitable	activation	contractility of cardiomyocytes↑	[91,92]
	mouse	cardiomyocytes	excitable	inhibition	Ca^2+^ overload↓	[93]
	rat	vascular smooth muscle cells	excitable	activation	vascular remodeling after injury, modulation of the cell migration	[94]
	mouse, human	vascular smooth muscle cells	excitable	inhibition	α1 adrenergic receptor↑, hypertension↓	[95]
Skeletal system	mouse	mature osteoclasts	non-excitable	activation	maturation and function of osteoclasts	[96]
	mouse	osteocytes	non-excitable	activation	bone homeostasis, regulation of Sost, Sp7, Tnfrsf11b	[97]
	mouse	osteoblasts	non-excitable	activation	osteoblastic differentiation, regulation of Alpl, Sp7, Dmp1 and Bglap	[98]
	mouse	osteoclast	non-excitable	activation	osteoclast differentiation	[99]
	mouse	chondrocytes	non-excitable	activation	major mechanical sensor	[79,100]
	mouse	annulus fibrosus cells	non-excitable	activation	regulator of perception of mechanical stimulation in intervertebral discs of spine	[101]
	human	synovial cells	non-excitable	activation	cell sensitivity↑ to noxious thermal changes and hypoosmotic stress	[102,103]
Nervous system	mouse	microglia	non-excitable	activation	microglia activation and proliferation, promoting functional and structural plasticity in excitatory spinal cord neurons via lipocalin-2	[104]
	rat	astrocytes	excitable	activation	promoting proliferation that protects neurons, initiates repair, and correlates with astrogliosis	[105]
	mouse	subpial endfeet of astrocytes	excitable	activation	hypoosmotic changes in cerebrospinal fluid	[106]
Reproductive system	mouse	oviduct	non-excitable	activation	ciliary beat frequency	[107]
	human	vaginal epithelial cells	non-excitable	activation	facilitating the entry of Herpes simplex virus (HSV)	[108]

## References

[1] White J.P., Cibelli M., Urban L., Nilius B., McGeown J.G., Nagy I. (2016). TRPV4: Molecular Conductor of a Diverse Orchestra. Physiol. Rev..

[2] Venkatachalam K., Montell C. (2007). TRP channels. Annu. Rev. Biochem..

[3] Moran M.M. (2018). TRP Channels as Potential Drug Targets. Annu. Rev. Pharmacol. Toxicol..

[4] Cosens D.J., Manning A. (1969). Abnormal electroretinogram from a Drosophila mutant. Nature.

[5] Montell C. (2005). The TRP superfamily of cation channels. Sci. STKE.

[6] Zhang M., Ma Y., Ye X., Zhang N., Pan L., Wang B. (2023). TRP (transient receptor potential) ion channel family: Structures, biological functions and therapeutic interventions for diseases. Signal Transduct. Target. Ther..

[7] Himmel N.J., Cox D.N. (2020). Transient receptor potential channels: Current perspectives on evolution, structure, function and nomenclature. Proc. Biol. Sci..

[8] Rosenbaum T., Islas L.D. (2023). Molecular Physiology of TRPV Channels: Controversies and Future Challenges. Annu. Rev. Physiol..

[9] Ho C.Y., Gu Q., Lin Y.S., Lee L.Y. (2001). Sensitivity of vagal afferent endings to chemical irritants in the rat lung. Respir. Physiol..

[10] Bohlen C.J., Priel A., Zhou S., King D., Siemens J., Julius D. (2010). A bivalent tarantula toxin activates the capsaicin receptor, TRPV1, by targeting the outer pore domain. Cell.

[11] Shibasaki K., Murayama N., Ono K., Ishizaki Y., Tominaga M. (2010). TRPV2 enhances axon outgrowth through its activation by membrane stretch in developing sensory and motor neurons. J. Neurosci..

[12] Nilius B., Biro T., Owsianik G. (2014). TRPV3: Time to decipher a poorly understood family member!. J. Physiol..

[13] Liedtke W. (2005). TRPV4 plays an evolutionary conserved role in the transduction of osmotic and mechanical stimuli in live animals. J. Physiol..

[14] Liedtke W., Choe Y., Martí-Renom M.A., Bell A.M., Denis C.S., Sali A., Hudspeth A.J., Friedman J.M., Heller S. (2000). Vanilloid receptor-related osmotically activated channel (VR-OAC), a candidate vertebrate osmoreceptor. Cell.

[15] Strotmann R., Harteneck C., Nunnenmacher K., Schultz G., Plant T.D. (2000). OTRPC4, a nonselective cation channel that confers sensitivity to extracellular osmolarity. Nat. Cell Biol..

[16] Delany N.S., Hurle M., Facer P., Alnadaf T., Plumpton C., Kinghorn I., See C.G., Costigan M., Anand P., Woolf C.J. (2001). Identification and characterization of a novel human vanilloid receptor-like protein, VRL-2. Physiol. Genom..

[17] Nilius B., Prenen J., Wissenbach U., Bödding M., Droogmans G. (2001). Differential activation of the volume-sensitive cation channel TRP12 (OTRPC4) and volume-regulated anion currents in HEK-293 cells. Pflug. Arch..

[18] Güler A.D., Lee H., Iida T., Shimizu I., Tominaga M., Caterina M. (2002). Heat-evoked activation of the ion channel, TRPV4. J. Neurosci..

[19] Laing R.J., Dhaka A. (2016). ThermoTRPs and Pain. Neuroscientist.

[20] Hong K., Cope E.L., DeLalio L.J., Marziano C., Isakson B.E., Sonkusare S.K. (2018). TRPV4 (Transient Receptor Potential Vanilloid 4) Channel-Dependent Negative Feedback Mechanism Regulates G(q) Protein-Coupled Receptor-Induced Vasoconstriction. Arterioscler. Thromb. Vasc. Biol..

[21] Luo J., Qian A., Oetjen L.K., Yu W., Yang P., Feng J., Xie Z., Liu S., Yin S., Dryn D. (2018). TRPV4 Channel Signaling in Macrophages Promotes Gastrointestinal Motility via Direct Effects on Smooth Muscle Cells. Immunity.

[22] Szabó I.L., Lisztes E., Béke G., Tóth K.F., Paus R., Oláh A., Bíró T. (2020). The Phytocannabinoid (-)-Cannabidiol Operates as a Complex, Differential Modulator of Human Hair Growth: Anti-Inflammatory Submicromolar versus Hair Growth Inhibitory Micromolar Effects. J. Investig. Dermatol..

[23] Szabó I.L., Herczeg-Lisztes E., Szegedi A., Nemes B., Paus R., Bíró T., Szöllősi A.G. (2019). TRPV4 Is Expressed in Human Hair Follicles and Inhibits Hair Growth In Vitro. J. Investig. Dermatol..

[24] She G., Hou M.C., Zhang Y., Zhang Y., Wang Y., Wang H.F., Lai B.C., Zhao W.B., Du X.J., Deng X.L. (2020). Gal-3 (Galectin-3) and K(Ca)3.1 Mediate Heterogeneous Cell Coupling and Myocardial Fibrogenesis Driven by βAR (β-Adrenoceptor) Activation. Hypertension.

[25] Zhan L., Li J. (2018). The role of TRPV4 in fibrosis. Gene.

[26] Rahaman S.O., Grove L.M., Paruchuri S., Southern B.D., Abraham S., Niese K.A., Scheraga R.G., Ghosh S., Thodeti C.K., Zhang D.X. (2014). TRPV4 mediates myofibroblast differentiation and pulmonary fibrosis in mice. J. Clin. Investig..

[27] Goswami R., Cohen J., Sharma S., Zhang D.X., Lafyatis R., Bhawan J., Rahaman S.O. (2017). TRPV4 ION Channel Is Associated with Scleroderma. J. Investig. Dermatol..

[28] Saez F., Hong N.J., Cabral P.D., Garvin J.L. (2020). Stretch-Induced Increases in Intracellular Ca Stimulate Thick Ascending Limb O_2_^−^) Production and Are Enhanced in Dahl Salt-Sensitive Rats. Hypertension.

[29] Greenstein A.S., Kadir S., Csato V., Sugden S.A., Baylie R.A., Eisner D.A., Nelson M.T. (2020). Disruption of Pressure-Induced Ca^2+^ Spark Vasoregulation of Resistance Arteries, Rather Than Endothelial Dysfunction, Underlies Obesity-Related Hypertension. Hypertension.

[30] Tang B., Wu J., Zhu M.X., Sun X., Liu J., Xie R., Dong T.X., Xiao Y., Carethers J.M., Yang S. (2019). VPAC1 couples with TRPV4 channel to promote calcium-dependent gastric cancer progression via a novel autocrine mechanism. Oncogene.

[31] Xie R., Xu J., Xiao Y., Wu J., Wan H., Tang B., Liu J., Fan Y., Wang S., Wu Y. (2017). Calcium Promotes Human Gastric Cancer via a Novel Coupling of Calcium-Sensing Receptor and TRPV4 Channel. Cancer Res..

[32] Mascarenhas N.L., Wang Z., Chang Y.L., Di Nardo A. (2017). TRPV4 Mediates Mast Cell Activation in Cathelicidin-Induced Rosacea Inflammation. J. Investig. Dermatol..

[33] Chen Y., Moore C.D., Zhang J.Y., Hall R.P., MacLeod A.S., Liedtke W. (2017). TRPV4 Moves toward Center-Fold in Rosacea Pathogenesis. J. Investig. Dermatol..

[34] Gonzalez-Lopez A., Lopez-Alonso I., Pickerodt P.A., von Haefen C., Amado-Rodriguez L., Reimann H., Niendorf T., Kuebler W., Albaiceta G.M., Francis R.C.E. (2019). Lung Purinoceptor Activation Triggers Ventilator-Induced Brain Injury. Crit. Care Med..

[35] Gorelick F., Nathanson M.H. (2020). TRPV4 helps Piezo1 put the squeeze on pancreatic acinar cells. J. Clin. Investig..

[36] Swain S.M., Romac J.M., Shahid R.A., Pandol S.J., Liedtke W., Vigna S.R., Liddle R.A. (2020). TRPV4 channel opening mediates pressure-induced pancreatitis initiated by Piezo1 activation. J. Clin. Investig..

[37] Deng Z., Paknejad N., Maksaev G., Sala-Rabanal M., Nichols C.G., Hite R.K., Yuan P. (2018). Cryo-EM and X-ray structures of TRPV4 reveal insight into ion permeation and gating mechanisms. Nat. Struct. Mol. Biol..

[38] Garcia-Elias A., Mrkonjic S., Pardo-Pastor C., Inada H., Hellmich U.A., Rubio-Moscardó F., Plata C., Gaudet R., Vicente R., Valverde M.A. (2013). Phosphatidylinositol-4,5-biphosphate-dependent rearrangement of TRPV4 cytosolic tails enables channel activation by physiological stimuli. Proc. Natl. Acad. Sci. USA.

[39] Cuajungco M.P., Grimm C., Oshima K., D’Hoedt D., Nilius B., Mensenkamp A.R., Bindels R.J., Plomann M., Heller S. (2006). PACSINs bind to the TRPV4 cation channel. PACSIN 3 modulates the subcellular localization of TRPV4. J. Biol. Chem..

[40] Inada H., Procko E., Sotomayor M., Gaudet R. (2012). Structural and biochemical consequences of disease-causing mutations in the ankyrin repeat domain of the human TRPV4 channel. Biochemistry.

[41] Nilius B., Watanabe H., Vriens J. (2003). The TRPV4 channel: Structure-function relationship and promiscuous gating behaviour. Pflug. Arch..

[42] Nilius B., Flockerzi V. (2014). Mammalian transient receptor potential (TRP) cation channels. Preface. Handb. Exp. Pharmacol..

[43] Garcia-Elias A., Lorenzo I.M., Vicente R., Valverde M.A. (2008). IP3 receptor binds to and sensitizes TRPV4 channel to osmotic stimuli via a calmodulin-binding site. J. Biol. Chem..

[44] Becker D., Müller M., Leuner K., Jendrach M. (2008). The C-terminal domain of TRPV4 is essential for plasma membrane localization. Mol. Membr. Biol..

[45] Strotmann R., Schultz G., Plant T.D. (2003). Ca^2+^-dependent potentiation of the nonselective cation channel TRPV4 is mediated by a C-terminal calmodulin binding site. J. Biol. Chem..

[46] Voets T., Prenen J., Vriens J., Watanabe H., Janssens A., Wissenbach U., Bödding M., Droogmans G., Nilius B. (2002). Molecular determinants of permeation through the cation channel TRPV4. J. Biol. Chem..

[47] Chaigne S., Barbeau S., Ducret T., Guinamard R., Benoist D. (2023). Pathophysiological Roles of the TRPV4 Channel in the Heart. Cells.

[48] Zhang M., Meng N., Wang X., Chen W., Zhang Q. (2022). TRPV4 and PIEZO Channels Mediate the Mechanosensing of Chondrocytes to the Biomechanical Microenvironment. Membranes.

[49] Lee W., Leddy H.A., Chen Y., Lee S.H., Zelenski N.A., McNulty A.L., Wu J., Beicker K.N., Coles J., Zauscher S. (2014). Synergy between Piezo1 and Piezo2 channels confers high-strain mechanosensitivity to articular cartilage. Proc. Natl. Acad. Sci. USA.

[50] Batan D., Peters D.K., Schroeder M.E., Aguado B.A., Young M.W., Weiss R.M., Anseth K.S. (2022). Hydrogel cultures reveal Transient Receptor Potential Vanilloid 4 regulation of myofibroblast activation and proliferation in valvular interstitial cells. FASEB J..

[51] Sharma S., Goswami R., Zhang D.X., Rahaman S.O. (2019). TRPV4 regulates matrix stiffness and TGFβ1-induced epithelial-mesenchymal transition. J. Cell Mol. Med..

[52] Al-Azzam N., Teegala L.R., Pokhrel S., Ghebreigziabher S., Chachkovskyy T., Thodeti S., Gavilanes I., Covington K., Thodeti C.K., Paruchuri S. (2020). Transient Receptor Potential Vanilloid channel regulates fibroblast differentiation and airway remodeling by modulating redox signals through NADPH Oxidase 4. Sci. Rep..

[53] Gombedza F., Kondeti V., Al-Azzam N., Koppes S., Duah E., Patil P., Hexter M., Phillips D., Thodeti C.K., Paruchuri S. (2017). Mechanosensitive transient receptor potential vanilloid 4 regulates Dermatophagoides farinae-induced airway remodeling via 2 distinct pathways modulating matrix synthesis and degradation. FASEB J..

[54] Adapala R.K., Katari V., Teegala L.R., Thodeti S., Paruchuri S., Thodeti C.K. (2021). TRPV4 Mechanotransduction in Fibrosis. Cells.

[55] Adapala R.K., Kanugula A.K., Paruchuri S., Chilian W.M., Thodeti C.K. (2020). TRPV4 deletion protects heart from myocardial infarction-induced adverse remodeling via modulation of cardiac fibroblast differentiation. Basic. Res. Cardiol..

[56] Ahn M.S., Eom Y.W., Oh J.E., Cha S.K., Park K.S., Son J.W., Lee J.W., Youn Y.J., Ahn S.G., Kim J.Y. (2020). Transient receptor potential channel TRPV4 mediates TGF-β1-induced differentiation of human ventricular fibroblasts. Cardiol. J..

[57] Parekh A.B. (2011). Decoding cytosolic Ca2+ oscillations. Trends Biochem. Sci..

[58] Janssen L.J., Farkas L., Rahman T., Kolb M.R. (2009). ATP stimulates Ca^2+^-waves and gene expression in cultured human pulmonary fibroblasts. Int. J. Biochem. Cell Biol..

[59] Dunn K.M., Hill-Eubanks D.C., Liedtke W.B., Nelson M.T. (2013). TRPV4 channels stimulate Ca2+-induced Ca2+ release in astrocytic endfeet and amplify neurovascular coupling responses. Proc. Natl. Acad. Sci. USA.

[60] Lv M., Zhou Y., Chen X., Han L., Wang L., Lu X.L. (2018). Calcium signaling of in situ chondrocytes in articular cartilage under compressive loading: Roles of calcium sources and cell membrane ion channels. J. Orthop. Res..

[61] Mukherjee S., Duan F., Kolb M.R., Janssen L.J. (2013). Platelet derived growth factor-evoked Ca^2+^ wave and matrix gene expression through phospholipase C in human pulmonary fibroblast. Int. J. Biochem. Cell Biol..

[62] Logan A., Pell V.R., Shaffer K.J., Evans C., Stanley N.J., Robb E.L., Prime T.A., Chouchani E.T., Cocheme H.M., Fearnley I.M. (2016). Assessing the Mitochondrial Membrane Potential in Cells and In Vivo using Targeted Click Chemistry and Mass Spectrometry. Cell Metab..

[63] Sack M.N. (2006). Mitochondrial depolarization and the role of uncoupling proteins in ischemia tolerance. Cardiovasc. Res..

[64] Zhang X., Lee M.D., Buckley C., Wilson C., McCarron J.G. (2022). Mitochondria regulate TRPV4-mediated release of ATP. Br. J. Pharmacol..

[65] Liang X., Xu S., Zhang J., Li J., Shen Q. (2018). Cascade Amplifiers of Intracellular Reactive Oxygen Species Based on Mitochondria-Targeted Core-Shell ZnO-TPP@D/H Nanorods for Breast Cancer Therapy. ACS Appl. Mater. Interfaces.

[66] Bhat E.A., Sajjad N. (2021). Human Pannexin 1 channel: Insight in structure-function mechanism and its potential physiological roles. Mol. Cell Biochem..

[67] Moesta A.K., Li X.Y., Smyth M.J. (2020). Targeting CD39 in cancer. Nat. Rev. Immunol..

[68] Domenici M.R., Ferrante A., Martire A., Chiodi V., Pepponi R., Tebano M.T., Popoli P. (2019). Adenosine A(2A) receptor as potential therapeutic target in neuropsychiatric disorders. Pharmacol. Res..

[69] Fernanda da Rocha L., Sérgio José Macedo J., Murilo Luiz C., Adair Roberto Soares S., Gowder S.G.T. (2014). Pharmacology of Adenosine Receptors and Their Signaling Role in Immunity and Inflammation. Pharmacology and Therapeutics.

[70] Shibasaki K., Sugio S., Takao K., Yamanaka A., Miyakawa T., Tominaga M., Ishizaki Y. (2015). TRPV4 activation at the physiological temperature is a critical determinant of neuronal excitability and behavior. Pflug. Arch..

[71] Shibasaki K., Suzuki M., Mizuno A., Tominaga M. (2007). Effects of body temperature on neural activity in the hippocampus: Regulation of resting membrane potentials by transient receptor potential vanilloid 4. J. Neurosci..

[72] Li L., Qu W., Zhou L., Lu Z., Jie P., Chen L., Chen L. (2013). Activation of Transient Receptor Potential Vanilloid 4 Increases NMDA-Activated Current in Hippocampal Pyramidal Neurons. Front. Cell Neurosci..

[73] Hong Z., Tian Y., Qi M., Li Y., Du Y., Chen L., Liu W., Chen L. (2016). Transient Receptor Potential Vanilloid 4 Inhibits gamma-Aminobutyric Acid-Activated Current in Hippocampal Pyramidal Neurons. Front. Mol. Neurosci..

[74] Qi M., Wu C., Wang Z., Zhou L., Men C., Du Y., Huang S., Chen L., Chen L. (2018). Transient Receptor Potential Vanilloid 4 Activation-Induced Increase in Glycine-Activated Current in Mouse Hippocampal Pyramidal Neurons. Cell Physiol. Biochem..

[75] Mukherjee S., Kolb M.R., Duan F., Janssen L.J. (2012). Transforming growth factor-β evokes Ca^2+^ waves and enhances gene expression in human pulmonary fibroblasts. Am. J. Respir. Cell Mol. Biol..

[76] Rahman M., Mukherjee S., Sheng W., Nilius B., Janssen L.J. (2016). Electrophysiological characterization of voltage-dependent calcium currents and TRPV4 currents in human pulmonary fibroblasts. Am. J. Physiol. Lung Cell Mol. Physiol..

[77] Shen J., Tu L., Chen D., Tan T., Wang Y., Wang S. (2019). TRPV4 channels stimulate Ca^2+^-induced Ca^2+^ release in mouse neurons and trigger endoplasmic reticulum stress after intracerebral hemorrhage. Brain Res. Bull..

[78] De Koninck P., Schulman H. (1998). Sensitivity of CaM kinase II to the frequency of Ca^2+^ oscillations. Science.

[79] Zhang M., Wu X., Du G., Chen W., Zhang Q. (2022). Substrate stiffness-dependent regulatory volume decrease and calcium signaling in chondrocytes. Acta Biochim. Biophys. Sin..

[80] Yarishkin O., Phuong T.T.T., Vazquez-Chona F., Bertrand J., van Battenburg-Sherwood J., Redmon S.N., Rudzitis C.N., Lakk M., Baumann J.M., Freichel M. (2022). Emergent Temporal Signaling in Human Trabecular Meshwork Cells: Role of TRPV4-TRPM4 Interactions. Front. Immunol..

[81] Che H., Yue J., Tse H.F., Li G.R. (2014). Functional TRPV and TRPM channels in human preadipocytes. Pflug. Arch..

[82] Gilchrist C.L., Leddy H.A., Kaye L., Case N.D., Rothenberg K.E., Little D., Liedtke W., Hoffman B.D., Guilak F. (2019). TRPV4-mediated calcium signaling in mesenchymal stem cells regulates aligned collagen matrix formation and vinculin tension. Proc. Natl. Acad. Sci. USA.

[83] Haywood N., Ta H.Q., Zhang A., Charles E.J., Rotar E., Noona S.T., Salmon M., Daneva Z., Sonkusare S.K., Laubach V.E. (2022). Endothelial Transient Receptor Potential Vanilloid 4 Channels Mediate Lung Ischemia-Reperfusion Injury. Ann. Thorac. Surg..

[84] Ottolini M., Hong K., Cope E.L., Daneva Z., DeLalio L.J., Sokolowski J.D., Marziano C., Nguyen N.Y., Altschmied J., Haendeler J. (2020). Local Peroxynitrite Impairs Endothelial Transient Receptor Potential Vanilloid 4 Channels and Elevates Blood Pressure in Obesity. Circulation.

[85] Hatano N., Itoh Y., Muraki K. (2009). Cardiac fibroblasts have functional TRPV4 activated by 4alpha-phorbol 12,13-didecanoate. Life Sci..

[86] Liao J., Wu Q., Qian C., Zhao N., Zhao Z., Lu K., Zhang S., Dong Q., Chen L., Li Q. (2020). TRPV4 blockade suppresses atrial fibrillation in sterile pericarditis rats. JCI Insight.

[87] Adapala R.K., Thoppil R.J., Luther D.J., Paruchuri S., Meszaros J.G., Chilian W.M., Thodeti C.K. (2013). TRPV4 channels mediate cardiac fibroblast differentiation by integrating mechanical and soluble signals. J. Mol. Cell Cardiol..

[88] Che H., Xiao G.S., Sun H.Y., Wang Y., Li G.R. (2016). Functional TRPV2 and TRPV4 channels in human cardiac c-kit(+) progenitor cells. J. Cell Mol. Med..

[89] Balducci V., Faris P., Balbi C., Costa A., Negri S., Rosti V., Bollini S., Moccia F. (2021). The human amniotic fluid stem cell secretome triggers intracellular Ca^2+^ oscillations, NF-κB nuclear translocation and tube formation in human endothelial colony-forming cells. J. Cell Mol. Med..

[90] Zou Y., Zhang M., Wu Q., Zhao N., Chen M., Yang C., Du Y., Han B. (2022). Activation of transient receptor potential vanilloid 4 is involved in pressure overload-induced cardiac hypertrophy. eLife.

[91] Peana D., Polo-Parada L., Domeier T.L. (2022). Arrhythmogenesis in the aged heart following ischaemia-reperfusion: Role of transient receptor potential vanilloid 4. Cardiovasc. Res..

[92] Veteto A.B., Peana D., Lambert M.D., McDonald K.S., Domeier T.L. (2020). Transient receptor potential vanilloid-4 contributes to stretch-induced hypercontractility and time-dependent dysfunction in the aged heart. Cardiovasc. Res..

[93] Jones J.L., Peana D., Veteto A.B., Lambert M.D., Nourian Z., Karasseva N.G., Hill M.A., Lindman B.R., Baines C.P., Krenz M. (2019). TRPV4 increases cardiomyocyte calcium cycling and contractility yet contributes to damage in the aged heart following hypoosmotic stress. Cardiovasc. Res..

[94] Li S.S., Gao S., Chen Y., Bao H., Li Z.T., Yao Q.P., Liu J.T., Wang Y., Qi Y.X. (2021). Platelet-derived microvesicles induce calcium oscillations and promote VSMC migration via TRPV4. Theranostics.

[95] Chen Y.L., Daneva Z., Kuppusamy M., Ottolini M., Baker T.M., Klimentova E., Shah S.A., Sokolowski J.D., Park M.S., Sonkusare S.K. (2022). Novel Smooth Muscle Ca^2+^-Signaling Nanodomains in Blood Pressure Regulation. Circulation.

[96] Li P., Bian X., Liu C., Wang S., Guo M., Tao Y., Huo B. (2018). STIM1 and TRPV4 regulate fluid flow-induced calcium oscillation at early and late stages of osteoclast differentiation. Cell Calcium.

[97] Williams K.M., Leser J.M., Gould N.R., Joca H.C., Lyons J.S., Khairallah R.J., Ward C.W., Stains J.P. (2020). TRPV4 calcium influx controls sclerostin protein loss independent of purinergic calcium oscillations. Bone.

[98] Suzuki T., Notomi T., Miyajima D., Mizoguchi F., Hayata T., Nakamoto T., Hanyu R., Kamolratanakul P., Mizuno A., Suzuki M. (2013). Osteoblastic differentiation enhances expression of TRPV4 that is required for calcium oscillation induced by mechanical force. Bone.

[99] Masuyama R., Vriens J., Voets T., Karashima Y., Owsianik G., Vennekens R., Lieben L., Torrekens S., Moermans K., Vanden Bosch A. (2008). TRPV4-mediated calcium influx regulates terminal differentiation of osteoclasts. Cell Metab..

[100] Du G., Li L., Zhang X., Liu J., Hao J., Zhu J., Wu H., Chen W., Zhang Q. (2020). Roles of TRPV4 and piezo channels in stretch-evoked Ca^2+^ response in chondrocytes. Exp. Biol. Med..

[101] Kim M.K., Ramachandran R., Séguin C.A. (2021). Spatiotemporal and functional characterisation of transient receptor potential vanilloid 4 (TRPV4) in the murine intervertebral disc. Eur. Cell Mater..

[102] Alessandri-Haber N., Dina O.A., Joseph E.K., Reichling D., Levine J.D. (2006). A transient receptor potential vanilloid 4-dependent mechanism of hyperalgesia is engaged by concerted action of inflammatory mediators. J. Neurosci..

[103] Kochukov M.Y., McNearney T.A., Yin H., Zhang L., Ma F., Ponomareva L., Abshire S., Westlund K.N. (2009). Tumor necrosis factor-alpha (TNF-alpha) enhances functional thermal and chemical responses of TRP cation channels in human synoviocytes. Mol. Pain..

[104] Hu X., Du L., Liu S., Lan Z., Zang K., Feng J., Zhao Y., Yang X., Xie Z., Wang P.L. (2023). A TRPV4-dependent neuroimmune axis in the spinal cord promotes neuropathic pain. J. Clin. Investig..

[105] Butenko O., Dzamba D., Benesova J., Honsa P., Benfenati V., Rusnakova V., Ferroni S., Anderova M. (2012). The increased activity of TRPV4 channel in the astrocytes of the adult rat hippocampus after cerebral hypoxia/ischemia. PLoS ONE.

[106] Eilert-Olsen M., Hjukse J.B., Thoren A.E., Tang W., Enger R., Jensen V., Pettersen K.H., Nagelhus E.A. (2019). Astroglial endfeet exhibit distinct Ca^2+^ signals during hypoosmotic conditions. Glia.

[107] Jung C., Fernández-Dueñas V., Plata C., Garcia-Elias A., Ciruela F., Fernández-Fernández J.M., Valverde M.A. (2018). Functional coupling of GABA(A/B) receptors and the channel TRPV4 mediates rapid progesterone signaling in the oviduct. Sci. Signal.

[108] Jiang P., Li S.S., Xu X.F., Yang C., Cheng C., Wang J.S., Zhou P.Z., Liu S.W. (2023). TRPV4 channel is involved in HSV-2 infection in human vaginal epithelial cells through triggering Ca^2+^ oscillation. Acta Pharmacol. Sin..

[109] Jiang D., Christ S., Correa-Gallegos D., Ramesh P., Kalgudde Gopal S., Wannemacher J., Mayr C.H., Lupperger V., Yu Q., Ye H. (2020). Injury triggers fascia fibroblast collective cell migration to drive scar formation through N-cadherin. Nat. Commun..

[110] Wan L., Jiang D., Correa-Gallegos D., Ramesh P., Zhao J., Ye H., Zhu S., Wannemacher J., Volz T., Rinkevich Y. (2021). Connexin43 gap junction drives fascia mobilization and repair of deep skin wounds. Matrix Biol..

[111] Rajendran V., Ramesh P., Dai R., Kalgudde Gopal S., Ye H., Machens H.G., Adler H., Jiang D., Rinkevich Y. (2023). Therapeutic Silencing of p120 in Fascia Fibroblasts Ameliorates Tissue Repair. J. Investig. Dermatol..

[112] Toumpanakis D., Chatzianastasiou A., Vassilakopoulou V., Mizi E., Dettoraki M., Perlikos F., Giatra G., Mikos N., Theocharis S., Vassilakopoulos T. (2022). TRPV4 Inhibition Exerts Protective Effects Against Resistive Breathing Induced Lung Injury. Int. J. Chron. Obs. Pulmon Dis..

[113] Nam M.H., Park H.J., Seo Y.K. (2023). Reduction of Osteoclastic Differentiation of Raw 264.7 Cells by EMF Exposure through TRPV4 and p-CREB Pathway. Int. J. Mol. Sci..

[114] Hurd L., Kirwin S.M., Boggs M., Mackenzie W.G., Bober M.B., Funanage V.L., Duncan R.L. (2015). A mutation in TRPV4 results in altered chondrocyte calcium signaling in severe metatropic dysplasia. Am. J. Med. Genet. A.

[115] O’Conor C.J., Ramalingam S., Zelenski N.A., Benefield H.C., Rigo I., Little D., Wu C.L., Chen D., Liedtke W., McNulty A.L. (2016). Cartilage-Specific Knockout of the Mechanosensory Ion Channel TRPV4 Decreases Age-Related Osteoarthritis. Sci. Rep..

[116] Cui Y.Y., Li M.Y., Li Y.T., Ning J.Y., Gou X.C., Shi J., Li Y.Q. (2020). Expression and functional characterization of transient receptor potential vanilloid 4 in the dorsal root ganglion and spinal cord of diabetic rats with mechanical allodynia. Brain Res. Bull..

[117] Jang Y., Jung J., Kim H., Oh J., Jeon J.H., Jung S., Kim K.T., Cho H., Yang D.J., Kim S.M. (2012). Axonal neuropathy-associated TRPV4 regulates neurotrophic factor-derived axonal growth. J. Biol. Chem..

[118] Rodrigues P., Ruviaro N.A., Trevisan G. (2022). TRPV4 Role in Neuropathic Pain Mechanisms in Rodents. Antioxidants.

[119] Jie P., Lu Z., Hong Z., Li L., Zhou L., Li Y., Zhou R., Zhou Y., Du Y., Chen L. (2016). Activation of Transient Receptor Potential Vanilloid 4 is Involved in Neuronal Injury in Middle Cerebral Artery Occlusion in Mice. Mol. Neurobiol..

[120] Liedtke W., Friedman J.M. (2003). Abnormal osmotic regulation in trpv4-/- mice. Proc. Natl. Acad. Sci. USA.

[121] Koide M., Wellman G.C. (2015). Activation of TRPV4 channels does not mediate inversion of neurovascular coupling after SAH. Acta Neurochir. Suppl..

